# COVID-19 and the Urgent Need for New Therapies for Obesity

**DOI:** 10.1089/pop.2020.0307

**Published:** 2021-10-01

**Authors:** Frank L. Greenway, Michelle Look, Angela Golden, Irfan Asif, Joe Nadglowski, Ted Kyle, Harry L. Leider

**Affiliations:** 1 Clinical Trials Unit, Pennington Biomedical Research Center, Baton Rouge, Louisiana, USA.; 2 Department of Family Medicine, Family Practice, Sports Medicine, Obesity Medicine, San Diego Sports Medicine and Family Health Center, San Diego, California, USA.; 3 NP from Home, LLC and NP Obesity Treatment Clinic, Munds Park, Arizona, USA.; 4 Department of Family and Community Medicine, University of Alabama Birmingham (UAB) School of Medicine, Birmingham, Alabama, USA.; 5 Obesity Action Coalition, Tampa, Florida, USA.; 6 ConscienHealth, Pittsburgh, Pennsylvania, USA.; 7 Gelesis, Inc., Boston, Massachusetts, USA.

**Keywords:** obesity, COVID-19, obesity and COVID-19 pandemics, population health, new therapies

Unfortunately, the COVID-19 pandemic continues globally. Growing evidence demonstrates that obesity is a strong predictor of intubation and death, especially in patients younger than age 65 years, and there is a hypothesis that this is related to increased inflammation caused by increased adipose tissue.^1^ On June 25, 2020, the Centers for Disease Control and Prevention added obesity, defined as a body mass index (BMI) ≥30kg/m^2^, to the list of underlying conditions that significantly increase the risk of severe illness in COVID-19 patients. While there are not yet studies demonstrating that reducing body weight prior to a COVID-19 infection, or a certain level of weight loss improves outcomes during a COVID infection, there is good reason to believe that weight loss is beneficial.

Obesity in the United States is part of a worldwide pandemic with approximately 40% of adults now having obesity. National Health and Nutrition Examination Survey data from 1999 to 2016 show the prevalence of obesity in men increased from 27% to 38%, and in women from 33% to 41%.^2^ Obesity increases the risk of many serious diseases and comorbidities including cardiovascular disease, diabetes, hypertension, depression, sleep apnea, cancers, and depression. Now there is an additional urgent need to rapidly address obesity at a population level because of its convergence with the COVID-19 pandemic.

The ACTION (Awareness, Care, and Treatment in Obesity maNagement) study, with more than 3000 participants, examined obesity-related perceptions, attitudes, and behaviors among people with obesity, as well as health care providers. The study showed that despite several weight loss attempts, only 10% of the population with obesity can lose and maintain significant long-term weight loss for at least 1 year using lifestyle approaches alone.^3^ Therefore, asking providers and patients to use lifestyle approaches, without support, will not address the core physiologic factors fueling obesity.

To address this major population health problem, therapies must meet the following criteria: efficacy in producing meaningful long-term weight management, an acceptable safety/tolerability profile, the ability to be prescribed long term, and wide accessibility. We did not focus on more expensive devices and bariatric surgery because of cost and higher risk, limiting their application to large populations.

## History of Obesity Therapies

In many ways the evolution of therapies for obesity parallels the history of therapies for another chronic condition – hypertension. In 1939, before there were effective medications for severe hypertension, a radical lifestyle intervention, the Rice diet, was used to control malignant hypertension. In the same decade, a surgical procedure (ie, sympathectomy) was used for essential hypertension. In the mid-1950s, medications including thiazide diuretics and reserpine were found to be efficacious. In the 1990s beta-blockers, calcium channel blockers, and angiotensin-converting enzyme (ACE) inhibitors were introduced for essential hypertension. This history is notable for the evolution of therapy from “lifestyle interventions,” (ie, diet) to surgery, to medications that affected the brain (ie, reserpine) or kidneys (ie, thiazides) and often had side effects, to medications that were targeted at the blood vessels and generally are very well tolerated (eg, ACE inhibitors).^4^ These medications are now broadly covered by payors.

Obesity therapies have a similar history. In 1862, Dr. William Harvey prescribed to William Banting a very low carbohydrate diet that successfully produced 46 pounds of weight loss in less than a year. Much later, in the 1960s and 1970s, a surgical therapy, ileojejunal bypass, was popular. More recently, diets such as the Ornish, Atkins, and keto diets have fallen in and out of favor. Weight loss diets often produce short-term weight loss; however, strong metabolic and hormonal changes occur during weight loss that work against long-term weight maintenance.^5^ Therefore, most people struggling with obesity require additional support and individual treatment plans.

## Therapies That Do Not Address the Underlying Physiology of Obesity

Based on this knowledge of weight loss physiology, we can conceptually divide obesity management therapies into ones that address obesity-related physiologic changes and those that do not, but provide varying levels of support. Interventions that do not directly address obesity-related changes in physiology include a rapidly growing number of digital apps. One popular digital platform is Noom, which uses artificial intelligence and human coaches to deploy cognitive behavioral therapy based on well-established diet and lifestyle principles. A study delivering the Diabetes Prevention Program (DPP) using Noom enrolled 43 adults with overweight or obesity and prediabetes in a 24-week virtual DPP.^6^ Weight loss was more than 5% at 24 weeks in 56% of starters (ie, patients who read 1 article per week in 4 of the first 16 weeks). These findings support the 24-week effectiveness of a mobile coaching tool that is scalable at a cost of $32.25 a month. Other digital platforms, typically offered through payors or employers, that report positive weight loss outcomes in diabetes prevention-type programs include Livongo, Lark, and Omada.

Millions of consumers use popular commercial diet plans. Many diet plans offer digital or telephonic coaching tools to supplement their legacy programs. In 2015, a systematic review evaluated the weight loss efficacy of 13 high-quality, randomized clinical trials (RTCs) of the 3 market leaders: Weight Watchers (WW), Jenny Craig (JC), and Nutrisystem.^7^ This review found that, using intent-to-treat analysis, each program produced the following results:

- Six RCTs compared WW with an educational intervention control group. One demonstrated 2.6% greater weight loss at 12 months, but comparison in 2 trials to behavior therapy produced mixed results. The cost of WW is approximately $14 per month for digital support, $26 per month with online workshops and digital support, and $38 per month for a program that includes personal coaching.
- One RCT compared JC with an educational control, and 2 compared JC with behavioral counseling support. All resulted in about 4.9% greater weight loss at 12 months. The JC program includes a supply of meals that costs $390 to $675 per month, depending on the number of meals provided.
- One RCT compared Nutrisystem to an educational program control, and 2 RTCs compared Nutrisystem to a behavioral counseling group. Nutrisystem resulted in a 3.8% greater weight loss than both control groups at 3 months, but no trial lasted for 12 months. Nutrisystem costs between $270 to $375 per month, depending on the number of meals provided.

The authors concluded that clinicians could consider referring patients with overweight or obesity to WW or JC, but other programs require additional long-term studies.

## Therapies That Address the Underlying Physiology of Obesity

Therapies that address obesity physiology include drug therapies and one Class II device. Current Food and Drug Administration-approved drugs for long-term use are orlistat, buproprion/naltrexone, phentermine/topiramate, and liraglutide. A 2018 review of major weight loss studies^8^ described a 5% or greater weight loss in 23% of subjects on placebo, 56% on phentermine, 72%–79% on phentermine-topiramate, 54%–81% on liraglutide, 39%–66% on naltrexone-bupropion, and 44% taking orlistat. High attrition rates of 30%–45% were seen in all of the trials. Most of these studies were 1–2 years in duration.

Although these drugs have efficacy for about half of the population, only about 2% of people in the United States with overweight or obesity receive pharmacotherapy.^9^ Multiple factors likely contribute to this low rate of prescribing. These include failure to recognize obesity as a disease, lack of training, suboptimal adherence rates, and limited insurance coverage. Also, many health care providers and their patients continue to believe that the primary treatment of obesity is lifestyle change. They also are sometimes concerned about the safety and tolerability of anti-obesity medications.

Similar to the early drugs for hypertension, current obesity drugs may not be “targeted” but act indirectly on the brain, or in the case of orlistat on fat absorption, therefore sometimes producing undesirable side effects. These side effects differ with each anti-obesity medication, but their presence limits the ability of these drugs to address the large population with overweight or obesity. Finally, another limitation of these drugs is that their indications are restricted to people with a BMI of 30kg/m^2^ or higher, or 27kg/m^2^ or higher in patients with obesity-related comorbidities.

Finally, the cost of these therapies can be problematic. Traditional Medicare coverage is not available, so Medicare beneficiaries must pay for these medications with discretionary income. There is growing, but variable, coverage for obesity drugs in Medicaid, health maintenance organizations, and commercial plans. In October of 2020, as listed on GoodRx, typical retail prices with coupons ranged from $137/month to $619/month for branded oral medications to almost $1288/month for liraglutide.

It is evident from the low prescription rates that available pharmacologic therapies for obesity have not solved the complex disease of obesity and the related overwhelming public health problem. As mentioned earlier, the COVID-19 pandemic exacerbates the need for additional approaches to treat overweight and obesity, because next to age, obesity is a major risk for poor outcomes from the COVID-19 pandemic. As illustrated by the history of therapies for hypertension, we also would benefit from more nonsystemic treatments, especially for patients with overweight (eg, BMIs of 25 to 30 kg/m^2^), where existing weight loss drugs are not indicated, except for patients with a BMI ≥27 kg/m2 who also have significant comorbidities.

One treatment indicated for the lower range of BMI (eg, ≥25 kg/m^2^), Plenity, is now available. Plenity is a novel Class II oral device with physiological mechanisms that are not systemic. It is a superabsorbent hydrogel (SAH) regulated as a device and was cleared by the FDA in mid-2019 for weight management in adults with a BMI of 25kg/m^2^ to 40kg/m^2^ when used with diet and exercise. This novel SAH is made from food-grade building blocks of carboxymethylcellulose and citric acid. When combined, these ingredients form SAH crystals that absorb 100 times their weight of water in the stomach. Three SAH capsules are taken 20 minutes before lunch and dinner with 16 ounces of water. This creates a low caloric density gel composed of 1–2 mm beads that fills about 25% of the stomach's volume. In the 24-week pivotal trial of 436 subjects, 59% of subjects lost more than 5% of their total body weight. Notably, those who lost 3% or more body weight at 8 weeks had an 85% likelihood of losing 10% of initial body weight at 24 weeks.^10^

The overall safety profile was not statistically different than placebo except for an overall increase in gastrointestinal adverse events. However, the increase in specific side effects such as abdominal distension, diarrhea, and constipation did not reach statistical significance and these side effects were mostly mild and transient. In addition, the FDA clearance did not restrict the duration of use of this SAH. In October of 2020, Plenity has a retail cost of $98 per month. It is contraindicated in patients who are pregnant or are allergic to cellulose, citric acid, sodium stearyl fumarate, gelatin, or titanium dioxide. The safety profile and indication for patients with a lower BMI makes this therapy a valuable addition to the current therapeutic options.

## Conclusion

The expanding armamentarium of obesity therapies provides needed new tools to address the public health problem of obesity. The convergence of the obesity and COVID-19 pandemics makes providing better access to this growing tool kit of therapies of urgent importance. No one tool or approach will be effective or desirable for everyone, so multiple therapies are needed and awareness regarding their availability is critical.

Also, increased knowledge of the value of long-term use of these therapies by providers and patients and major changes at the policy and payer levels are critical. There is an urgent need to demonstrate cost savings of weight loss treatments to our health care system. These data will encourage broader coverage of evidence-based obesity therapies. As occurred with hypertension, newer therapies and tools are emerging that meet population health criteria of efficacy, safety/tolerability, ability to be used long term, and accessibility. This will drive a compelling argument for broader coverage by payors. Even though we now have effective vaccines for COVID-19, it will be many months until we produce herd immunity; even then, there still will be a critical need to address the worsening obesity pandemic.
